# Permethrin resistance in *Aedes aegypti*: Genomic variants that confer knockdown resistance, recovery, and death

**DOI:** 10.1371/journal.pgen.1009606

**Published:** 2021-06-17

**Authors:** Karla Saavedra-Rodriguez, Corey L. Campbell, Saul Lozano, Patricia Penilla-Navarro, Alma Lopez-Solis, Francisco Solis-Santoyo, Americo D. Rodriguez, Rushika Perera, William C. Black IV

**Affiliations:** 1 Colorado State University, Department of Microbiology, Immunology and Pathology, Center of Vector-borne and Infectious Diseases, Fort Collins, Colorado, United States of America; 2 Centers for Diseases Prevention and Control, Arboviral Diseases Branch, Fort Collins, Colorado, United States of America; 3 Centro Regional de Investigacion en Salud Publica, Instituto Nacional de Salud Publica, Tapachula, Mexico; The University of North Carolina at Chapel Hill, UNITED STATES

## Abstract

Pyrethroids are one of the few classes of insecticides available to control *Aedes aegypti*, the major vector of dengue, chikungunya, and Zika viruses. Unfortunately, evolving mechanisms of pyrethroid resistance in mosquito populations threaten our ability to control disease outbreaks. Two common pyrethroid resistance mechanisms occur in *Ae*. *aegypti*: 1) knockdown resistance, which involves amino acid substitutions at the pyrethroid target site—the voltage-gated sodium channel (VGSC)—and 2) enhanced metabolism by detoxification enzymes. When a heterogeneous population of mosquitoes is exposed to pyrethroids, different responses occur. During exposure, a proportion of mosquitoes exhibit immediate knockdown, whereas others are not knocked-down and are designated knockdown resistant (kdr). When these individuals are removed from the source of insecticide, the knocked-down mosquitoes can either remain in this status and lead to dead or recover within a few hours. The proportion of these phenotypic responses is dependent on the pyrethroid concentration and the genetic background of the population tested. In this study, we sequenced and performed pairwise genome comparisons between kdr, recovered, and dead phenotypes in a pyrethroid-resistant colony from Tapachula, Mexico. We identified single-nucleotide polymorphisms (SNPs) associated with each phenotype and identified genes that are likely associated with the mechanisms of pyrethroid resistance, including detoxification, the cuticle, and insecticide target sites. We identified high association between kdr and mutations at *VGSC* and moderate association with additional insecticide target site, detoxification, and cuticle protein coding genes. Recovery was associated with cuticle proteins, the voltage-dependent calcium channel, and a different group of detoxification genes. We provide a list of detoxification genes under directional selection in this field-resistant population. Their functional roles in pyrethroid metabolism and their potential uses as genomic markers of resistance require validation.

## Author summary

Dengue, Zika, and chikungunya are viral diseases transmitted by the mosquito *Aedes aegypti*. Unfortunately, no vaccines or effective treatments are available, and public health strategies rely on the suppression of mosquito populations to reduce the impact of disease outbreaks. Pyrethroids are a common class of insecticides used to suppress mosquito populations. Unfortunately, mosquitoes have developed resistance to pyrethroids in several regions of the world, threatening disease control efforts. Since few classes of insecticides are available for public health, we have to find strategies that prevent or prolong the use of pyrethroids. Therefore, we need to understand mechanisms of resistance and develop methods to quantify these mechanisms in field populations before they become a threat to vector control strategies. Quantifying the frequency of mutations associated with target site resistance provides information about the presence of this mechanism in mosquito populations; however, we still need to understand additional mechanisms of resistance (e.g., metabolism, cuticle penetration) that play an important role in the survival of mosquitoes. In this study, we sequenced the genome of resistant (kdr), recovered, and dead mosquitoes after insecticide exposure. Our aim is to identify genomic variants associated with specific mechanisms of resistance. The results will improve the identification of specific genomic markers associated with resistance in the *Aedes aegypti* from Southeastern Mexico.

## Introduction

The mosquito *Aedes aegypti* is the primary urban vector of three globally important arboviral diseases—dengue, Zika, and chikungunya fever—for which vaccines and effective pharmaceuticals are still lacking. The only available strategy to suppress these arboviral outbreaks is to reduce vector populations. Control of *Ae*. *aegypti* is challenging and is further compromised by widespread pyrethroid resistance [1–4]. Heavy use of pyrethroid space sprays—due to their strong human safety profile—has created immense selection pressure for resistance [1–4]. This resistance is primarily under the control of the voltage-gated sodium channel (VGSC) and enhanced metabolism by detoxification enzymes.

Amino acid replacements at VGSC—the target site of pyrethroids—confer resistance to knockdown (*kdr*-mutations) [4, 5]. Approximately 12 nonsynonymous substitutions in the VGSC gene (*VGSC*) are associated with pyrethroid resistance in *Ae*. *aegypti*. Three *kdr*-mutations are common in Latin America, including V410L, V1016I, and F1534C [6–8]. The role of V410L and F1534C to confer pyrethroid resistance was recently confirmed in electrophysiological assays [8, 9]. Although V1016I alone had no effect on the channel sensitivity to permethrin or deltamethrin, it enhanced the F1534C-mediated resistance to both pyrethroids [10].

Additional mechanisms of pyrethroid resistance are conferred by enhanced insecticide metabolism (or sequestration) by three enzyme systems, the carboxyl/choline/esterases (CCE), glutathione-s-transferases (GST), and cytochrome P450 monooxygenases (CYP) [11]; reduced penetration of insecticides through the cuticle [12]; and behavioral avoidance [13]. The extent to which these different mechanisms contribute to the overall resistance phenotype seems to vary [5, 6, 14, 15]. Previous studies in *Ae*. *aegypti* from Mexico showed two major quantitative trait loci (QTLs) controlling permethrin resistance [5]. One corresponded to the nonsynonymous mutation V1016I in the *VGSC* and the esterase *CCEunk70*. Additional QTLs contained several CYP genes of relatively minor effect. These results confirmed that target site insensitivity explained almost 60% of permethrin resistance but that other genes dispersed throughout the genome also contributed to the survival of mosquitoes following permethrin exposure.

A common methodology to diagnose pyrethroid resistance in mosquito populations is the bottle bioassay [16]. The major route of intoxication is through tarsal contact during exposure times that range from 30 min to 2 h, depending on the pyrethroid concentration. At the endpoint of the bioassay, two phenotypes are discriminated: knockdown or resistant mosquitoes. Intriguingly, once the knocked-down mosquitoes are removed from insecticide exposure, recovery rates from 20 to 60% have been reported in the literature [17]. Previous studies have shown that kdr mosquitoes often carry resistant homozygous genotypes at three loci in *VGSC* (V410L, V1016I, and F1534C). In contrast, recovered and dead mosquitoes often carry heterozygous or wild-type homozygous genotypes at these loci [5, 7, 15]. Two different studies showed that 42 and 32% of the recovered mosquitoes carry the V1016I heterozygous genotype [5, 15], suggesting partial protection during recovery, however, the remaining recovered individuals (>58%) must rely in mechanisms other than *kdr-*mutations to recover.

In this study, we aim to identify genomic differences associated with kdr, recovered, and dead phenotypes in mosquitoes following permethrin exposure in the laboratory. We used a F_1_ offspring of a pyrethroid-resistant *Ae*. *aegypti* field population from Tapachula, Mexico. Our hypothesis is that SNPs associated with kdr will occur at insecticide target site or cuticle genes, whereas recovery will be associated with genes linked to insecticide detoxification mechanisms. The identification of such SNPs will improve our understanding of the mechanisms of resistance associated with kdr, recovered, and dead in field pyrethroid-resistant mosquito populations.

## Results

**Fig 1:**
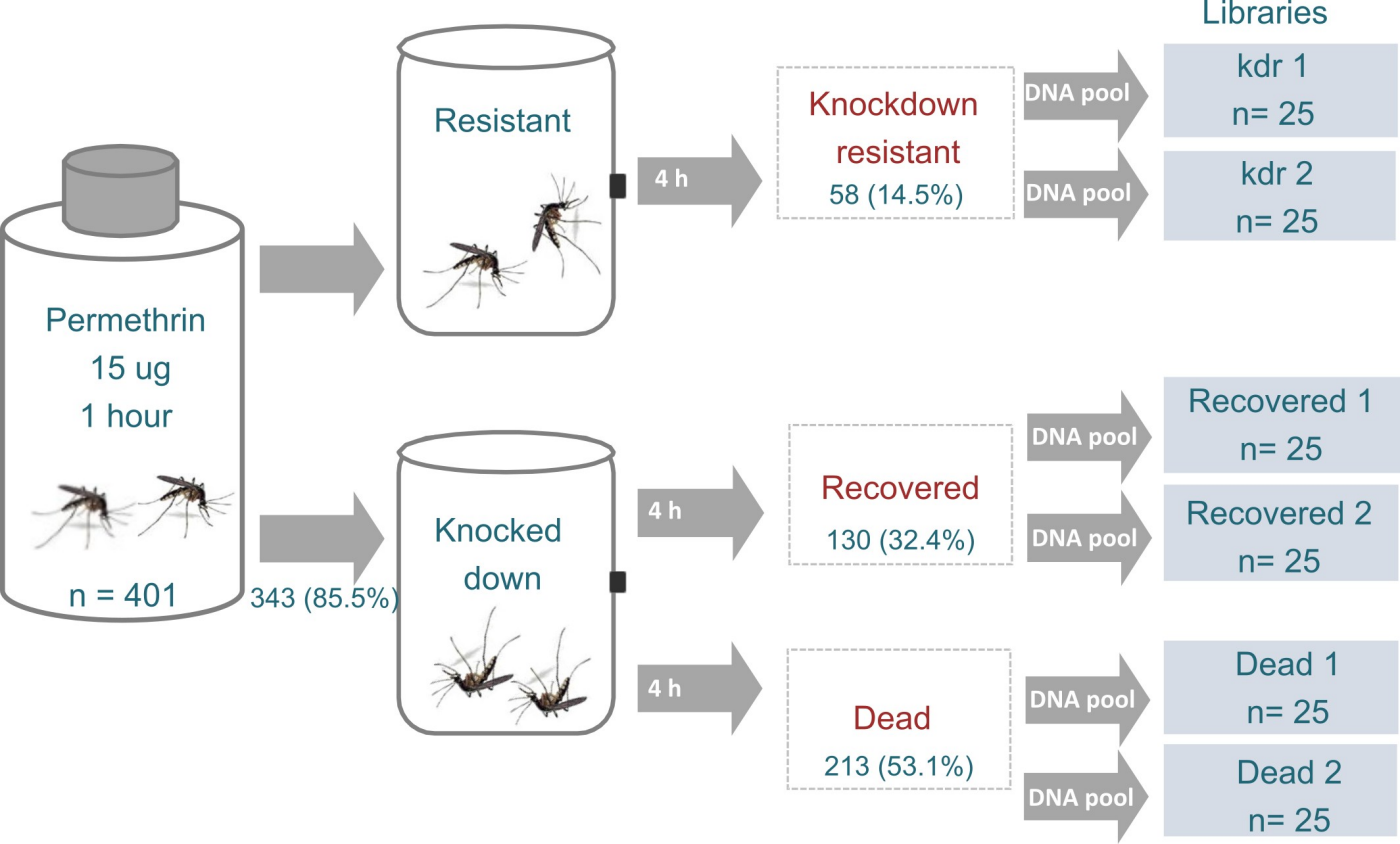
Bioassay to differentiate three phenotypes in *Aedes aegypti* exposed to permethrin (15 ug/bottle) for 1 h. Total number of mosquitoes used in bioassays are shown. Pooled libraries were prepared using 25 individual mosquitoes from each phenotypic group.

We exposed 401 mosquitoes for 1 h in bottles coated with 15 μg of permethrin. This permethrin concentration allowed us to discriminate three different phenotypes with significant sample size in a heterogeneous pyrethroid-resistant population from Tapachula. Fig 1 shows the three phenotypes discriminated by this concentration and time of exposure: 1) kdr (n = 58, 14.5% of total), 2) recovered (n = 130, 32.4% of total), and 3) dead (n = 213, 53.1% of total).

Six genomic libraries, consisting of two biological replicates of pooled mosquitoes (n = 25) exhibiting the kdr, recovered, or dead phenotypes were prepared. The cost of library enrichment using an exome-capture hybridization prevented us to process a third biological replicate of each phenotype. By using two replicates, we obtained between 112 and 129 million reads across the six pair-end libraries. Thus, sequencing coverage ranged from 196-fold to 288-fold. After removing repetitive DNA (coverage > 1000) and sites with fewer than 25 reads, we identified 30–35 million common sites among the three pairwise comparisons: 1) kdr vs recovered, 2) recovered vs dead, and 3) kdr vs dead. Between 1.69 and 2.3 million polymorphic sites (SNPs) were identified among the pairwise comparisons. Alternate nucleotides were defined as those differing from the reference genome. The frequency of the alternate nucleotide at each SNP was subjected to a contingency χ^2^ analysis and then assigned a genetic association value (-log_10_(*p* value)), referred to as the “LOD”. A Benjamini-Hochberg correction [18] for false discovery rate (FDR) was applied to SNPs at each chromosome separately using an α = 0.01. The LOD cutoff values ranged from 3.17 to 3.37 between the pairwise comparisons, resulting in 12,209 significant SNPs in the kdr vs recovered, 11,472 in the recovered vs dead, and 13,011 in the kdr vs dead comparison. The minimum and maximum LODs were 3.1 and 37, respectively. The mean LODs among SNPs ranged from 5.1 to 5.4, and the 95 quantiles, from 8.5 to 9.18 (Table 1). Approximately 55% of these SNPs occurred at intergenic sites, 7.2% at 3’-UTR sites, 7.5% at 5’-UTR sites, 36.9% at synonymous coding sites, 9.6% at nonsynonymous coding sites, 37.8% at intron sites, and 0.6% in noncoding RNA.

**Table 1:** Mean and standard error (SE) of LOD values (-log_10_(*p value*)) assigned to SNPs differing between kdr, recovered and dead *Aedes aegypti* exposed to permethrin. The number of SNPs (N) and the LOD mean and SE for three categories of genes associated with insecticide resistance are shown separately.

	kdr vs Recovered			Recovered vs Dead			kdr vs Dead		
	N	Mean	SE	N	Mean	SE	N	Mean	SE
**All**	12209	5.150	0.017	11472	5.112	0.016	13011	5.414	0.020
95 quantile	612	8.780		577	8.518		519	9.189	
**By category**									
Cuticle	88	5.010	0.149	112	5.102	0.139	138	5.394	0.168
Detoxification	259	4.757	0.084	280	4.930	0.088	340	5.190	0.082
Target sites	192	8.696	0.410	89	5.191	0.204	128	14.379	0.894
Other	11670	5.101	0.016	10991	5.116	0.017	12405	5.328	0.017

The mean LODs for SNPs belonging to three gene categories associated with insecticide resistance (cuticle, detoxification, and insecticide target sites) are shown in Table 1. For the cuticle category, the mean LODs were not significantly different between the phenotypes (F = 1.67, *p* value = 0.18). However, the mean LODs were significantly different for the target site category (F = 50.5, *p* value = 2e-16), in which the kdr vs recovered and kdr vs dead had mean LODs of 8.69 and 14.3, respectively, while the recovered vs dead comparison had a mean LOD of 5.19. In the detoxification category, we found significant differences between mean LODs (F = 6.76, *p* value = 0.001), with the kdr vs recovered and kdr vs dead explaining this difference.

**Fig 2:**
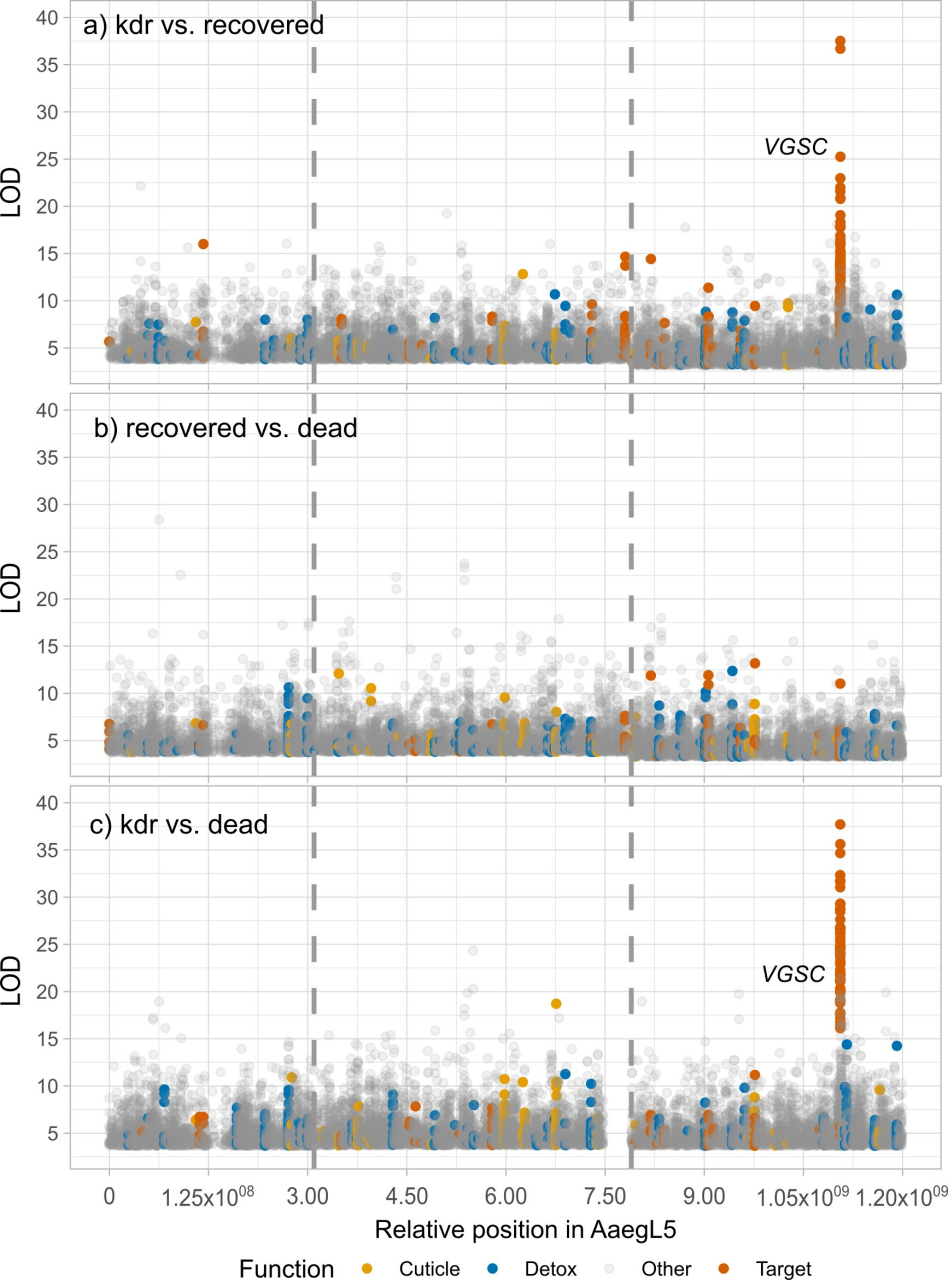
Distribution of SNPs associated with kdr, recovered, and dead *Aedes aegypti* exposed to permethrin. The relative physical position is based in *Ae*. *aegypti* AaegL5 assembly. LODs correspond to the–log_10_(*p value*) obtained in a chi square test that compared the proportion of the alternate allele between pairwise comparisons. A) kdr vs recovered and B) recovered vs dead and C) kdr vs dead. SNPs located in insecticide target site (yellow), detoxification (red), and cuticle genes (green) are highlighted.

The distributions of the SNPs by LOD and relative physical position across chromosomes for each of the pairwise comparisons are shown in Fig 2. Interestingly, the kdr vs recovered and the kdr vs dead comparisons showed a cluster of SNPs with high association values (> 95 quantiles) in chromosome 3 (Fig 2A and 2C). These clusters consist of SNPs in *VGSC*, the major pyrethroid target site. In contrast, the recovered vs dead comparison lacked this cluster of SNPs (Fig 2B), validating the role of VGSC mutations in kdr but not in recovery.

### SNPs that differ between kdr and recovery

The mosquitoes included in this comparison survived the exposure to permethrin; however, some mosquitoes exhibited knockdown resistance at 1 h of exposure (kdr), whereas others survived by recovering from initial knockdown during the 4-h observation. A total of 12,209 significant SNPs resulted in this comparison (2242, 4520, and 5447 in chromosomes 1, 2, and 3, respectively). Fig 2A shows the distribution of SNPs by their LOD values and relative positions in the genome. We purposely highlighted the SNPs associated with insecticide target site, detoxification, and cuticle genes.

### Top nonsynonymous SNPs associated with kdr

In this section, we describe nonsynonymous mutations while assuming that these mutations confer changes in protein activity or functionality, therefore, making these proteins more likely to be subject to selection. S1 Table shows the list of nonsynonymous SNPs associated with kdr. On chromosome 1, 195 nonsynonymous mutations were located in 149 genes. The SNPs with the highest LODs were D765E (LOD = 22.15) and N772K (LOD = 10.84) in the ER degradation-enhancing alpha-mannosidase (LOC5566778). These were followed by P194S (LOD = 10.72) in the bis(5’-adenosyl)-triphosphatase ENPP4 (LOC5572000). In chromosome 2, 463 nonsynonymous mutations in 295 genes were identified (S1 Table). The SNP with the highest LOD was R577S (LOD = 13.34) in a zinc finger protein 883 (LOC5564538), followed by Q773P (LOD = 12.06) in an activating transcription factor 7-interacting protein (LOC5569161) and D875E (LOD = 11.79) in the fatty acid synthase (LOC5573930). In chromosome 3, 517 nonsynonymous mutations in 336 genes were significantly associated with kdr (S1 Table). The SNPs with the highest LODs were S679T (LOD = 13.7) and V408L (LOD = 12.25) at *VGSC* (LOC5567355). These SNPs correspond to loci V723 and V410L following the *M*. *domestica* annotation. Additional SNPs were H1711P (LOD = 12.08) and K1391R (LOD = 10.76), located in uncharacterized genes LOC5571908 and LOC5572722, respectively.

### Insecticide target-site SNPs associated with kdr

We identified 192 significant SNPs in 23 genes coding for insecticide target sites; 70 SNPs were found only in *VGSC*. Table 2 shows the eight nonsynonymous SNPs identified in four genes, including acetylcholinesterase/hydrolase (*ACE*, LOC5570776), gamma-aminobutyric acid type B receptor subunit 2 (*GPRGBB3*, LOC5569525), G protein-activated inward rectifier potassium channel (*KCNJ3*, LOC5571228), and *VGSC* (LOC5567355*)*. The highest LODs occurred in S679T and V410L in the *VGSC* (LOD = 13.7 and 12.25), followed by *GPRGBB3* (LOD = 8.68 and 5.5) and *ACE* (LOD = 5.29 and 4.22).

**Table 2:** Nonsynonymous SNPs at insecticide target sites genes associated with kdr, recovered, or dead *Aedes aegypti* exposed to permethrin. Gene identification, chromosome (Ch), SNP site and amino acid residue (based in the AaegL5 genome assembly), LOD = -log_10_(*p* value vector base gene identification (from www.vectorbase.org). Voltage-gated calcium channel (*VGCC*), voltage-gated sodium channel (*VGSC)*, gamma-aminobutyric acid type B receptor subunit 2 (*GPRGBB3*), acetylcholinesterase/hydrolase (*ACE*), and G protein-activated inward rectifier potassium channel 3 (*KCNJ3*).

Gene	Gene ID	Ch	Site	Residue	LOD	Vector Base ID
**kdr vs recovered**						
*VGSC*	LOC5567355	3	316014588	S679T	13.74	AAEL006019
			316080722	V410L	12.25	
*GPRGBB3*	LOC5569525	2	470687501	G18V	8.38	AAEL007709
			470687506	T20P	5.50	
*ACE*	LOC5570776	3	29508519	L113S	5.29	AAEL008532
			29508507	Q117P	4.22	
*KCNJ3*	LOC5571228	3	50262691	S471N	3.69	AAEL013373
			50262715	G479V	3.28	
**Recovered vs dead**						
*VGCC*	LOC5564339	3	116602456	P1661S	10.92	AAEL004201
*GPRGBB3*	LOC5569525	2	470687501	G18V	7.62	AAEL007709
			470687506	T20P	7.13	
acetylcholine receptor	LOC5575838	1	44469	K297I	6.76	AAEL012106
*VGSC*	LOC5567355	3	316080722	V408L	4.62	AAEL006019
			316014588	S679T	3.88	
**Kdr vs dead**						
*VGSC*	LOC5567355	3	316014588	S679T	26.11	AAEL006019
			316080722	V410L	25.73	
*GPRGBB3*	LOC5569525	2	470713597	D963G	5.30	AAEL007709
			470713596	S963G	5.14	
*ACE*	LOC5570776	3	29508519	L113S	5.29	

**Fig 3:**
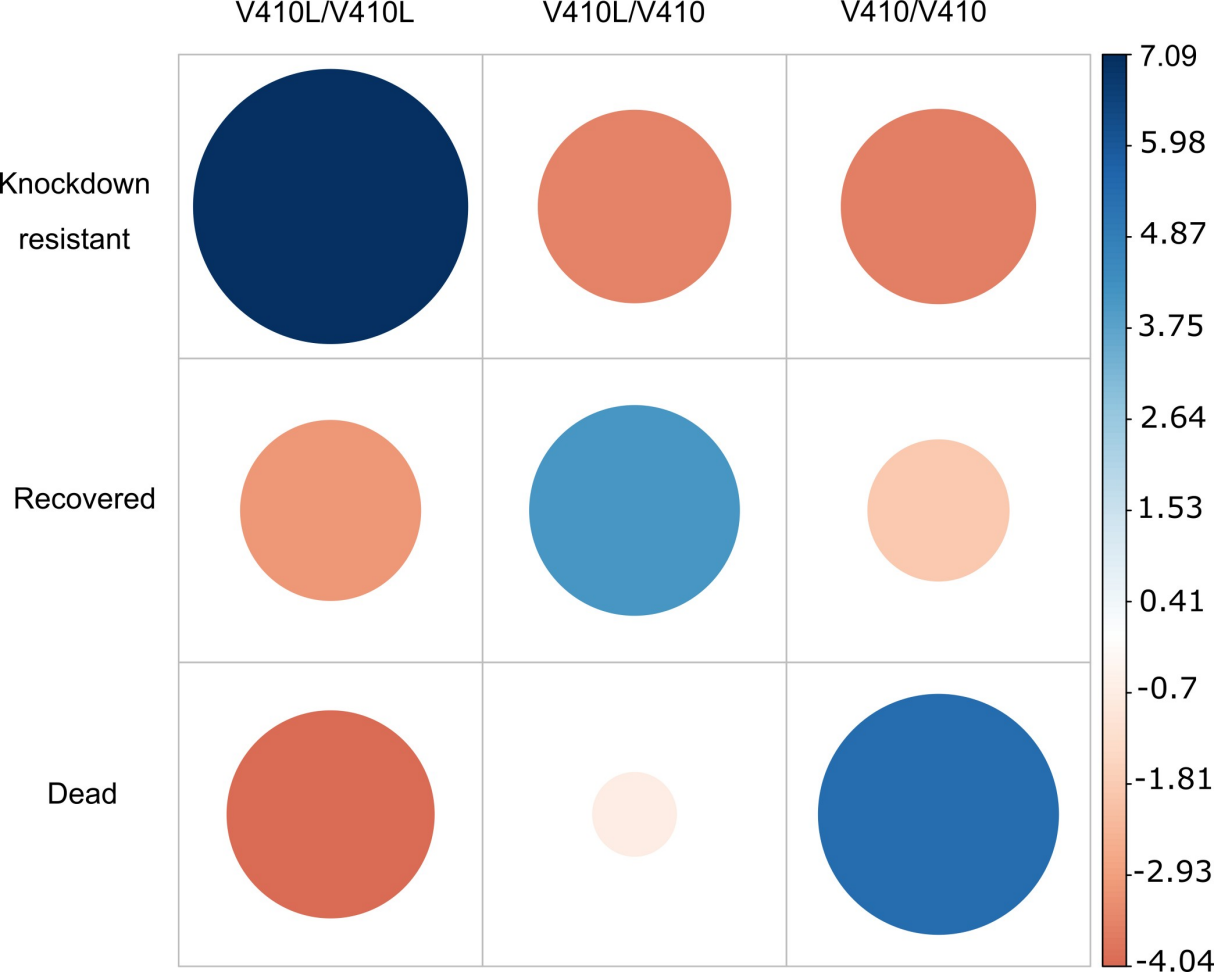
Pearson residuals between three genotypes at V410L in *VGSC* and the three phenotypes: kdr, recovered, and dead in *Aedes aegypti* mosquitoes exposed to permethrin. Genotypes were determined by an allele-specific PCR to detect three genotypes: homozygote resistant = V410L/V410L, heterozygote = V410L/V410), and wild-type homozygote = V410/V410. Positive residuals in blue specify a positive association between the corresponding row and column variables. Negative residuals are in red, implying a negative association between the corresponding row and column variables.

The 50 mosquitoes used to generate the kdr, recovered, and dead libraries were individually genotyped for V410L using an allele-specific PCR. Ninety percent of the kdr mosquitoes were resistant homozygotes (V410L/V410L), whereas 10% were heterozygotes (V410L/V410), and 0% were wild-type homozygotes (V410/V410). In contrast, 8% of the recovered were resistant homozygotes, 80% were heterozygotes, and 12% were wild-type homozygotes. None of the dead mosquitoes were resistant homozygotes, 36% were heterozygotes, and 64% were wild-type homozygotes at this locus. A 3 x 3 contingency table showed significant differences between observed and expected genotypes at each phenotype (χ^2^ = 168.8, df = 4 and *p* value = 1.8e-35). Fig 3 shows the Pearson residuals between the phenotypes and genotypes at locus V410L. Positive residuals in blue indicate a positive association between kdr and V410L/V410L and dead with V410/V410. Interestingly, recovery was positively associated with heterozygotes (V410L/V410).

### Cuticle SNPs associated with kdr

Changes in the cuticle proteins can result in reduced penetration by insecticides. Approximately 88 SNPs were identified in this category. The eight nonsynonymous SNPs located in seven genes are shown in Table 3. The highest LODs occurred in cuticle protein CP14.6 (LOC5577605, LOD = 6.46), in an uncharacterized cuticle protein (LOC5572415, LOD = 6.02), and in cuticle protein 8 (LOC5565392, LOD = 5.89).

**Table 3:** Nonsynonymous SNPs in cuticular protein genes associated with kdr, recovered, or dead *Aedes aegypti* exposed to permethrin. Gene identification, chromosome (Ch), SNP site, amino acid residue (based in the AaegL5 genome assembly), LOD = -log_10_(*p* value) and vector base identification (www.vectorbase.org).

Gene	Gene ID	Ch	Site	Residue	LOD	Vector Base ID
**kdr vs recovered**						
cuticle protein CP14.6	LOC5577605	2	366556230	T7A	6.46	AAEL003274
cuticle protein	LOC5572415	1	276097952	I229V	6.02	AAEL009783
			276097955	L228I	5.54	
cuticle protein 8	LOC5565392	2	288617520	I170V	5.89	AAEL004749
adult cuticle protein 1	LOC5573913	2	429075334	A75T	5.70	AAEL002191
cuticle protein CP14.6	LOC5570386	2	67628247	I34V	5.30	AAEL008284
cuticle protein 19	LOC5571330	2	178019830	D59N	3.97	AAEL008984
larval cuticle protein A2B	LOC5573609	3	177543253	S55R	3.69	AAEL002099
**Recovered vs dead**						
larval cuticle protein A2B	LOC5577372	3	186525449	Q84E	8.88	AAEL000419
			186525538	Y54C	6.30	
			186525518	S61A	5.11	
endocuticle SgAbd-6	LOC5570308	3	6190598	T112S	5.82	AAEL008251
cuticle protein	LOC5572416	1	276091198	I122V	5.58	AAEL009791
			276091186	A126T	3.91	
endocuticle SgAbd-5	LOC5568889	2	318779482	A14V	5.57	AAEL007194
cuticle protein 19	LOC5571355	2	178378901	A14V	4.10	AAEL008979
larval cuticle protein LCP-30	LOC5577596	2	366431631	K223N	4.00	AAEL003266
cuticle protein 19	LOC5571330	2	178019830	D59N	3.97	AAEL008984
cuticle protein 6	LOC5572360	3	284327757	P95A	3.85	AAEL009752
flexible cuticle protein 12	LOC110679003	3	127977229	G49S	3.45	AAEL002458
**kdr vs dead**						
adult cuticle protein 1	LOC5573913	2	429075334	A75T	6.58	AAEL002191
endocuticle SgAbd-6	LOC5570308	3	6190636	H99R	5.85	AAEL008251
cuticle protein CP14.6	LOC5575766	2	14463512	I16V	5.23	AAEL002725
pupal cuticle protein 36a	LOC5565726	3	218238646	P304A	5.09	AAEL004951
larval cuticle protein A2B	LOC5577372	3	186525449	Q84E	4.93	AAEL000419
			186525538	Y54C	4.93	
pupal cuticle protein 36	LOC5577597	2	366494266	A195T	4.70	AAEL003241
cuticle protein CP14.6	LOC5577605	2	366556230	T7A	4.69	AAEL003274
cuticle protein 8	LOC5565392	2	288617520	I170V	4.56	AAEL004749
larval cuticle protein A2B	LOC5565377	2	288426029	Q23*	4.44	AAEL004752
larval cuticle protein A2B	LOC5565357	2	288342641	A150T	4.31	AAEL004760
larval cuticle protein A2B	LOC5565394	2	288444286	E32Q	4.04	AAEL004772
larval cuticle protein LCP-30	LOC5577603	2	366503144	N33K	3.77	AAEL003222

### Detoxification SNPs associated with kdr

Two hundred ninety-five SNPs located in 91 genes differed significantly in the kdr vs recovered comparison. The 41 nonsynonymous SNPs were located in 31 detoxification genes (Table 4). In chromosome 1, the highest association occurred in *CYP6AL3* (LOD = 7.57), *GSTD3* (LOD = 7.27), and *CYP4C51* (LOD = 5.18). In chromosome 2, the highest association occurred in *CYP4AR2* (LOD = 9.4), *CYP6N15* (LOD = 5.3), and aldo-keto reductase (LOD = 5.2). In chromosome 3, the highest association occurred in *CYP285A* (LOD = 5.29), *CYP325U1* (LODs = 5.27, 5.22), and in the esterases *CCEunk7O* (LOD = 4.8) and *CCEbe1O* (LOD = 4.25).

**Table 4:** Nonsynonymous SNPs in detoxification genes associated with *kdr*, recovered, or dead *Aedes aegypti* exposed to permethrin. The gene identification, site, amino acid residue (based in the AaegL5 genome assembly), frequency of alternative nucleotide for each phenotype, the associated LOD values, chromosome (Ch) and vector base identification (www.vectorbase.org) are shown. * indicates SNPs occurring in two or more pairwise comparisons.

Gene		Gene ID	Site	Residue	Frequency		LOD	Ch	Vector Base ID
kdr vs recovered					kdr	recovered			
*CYP4AR2*		LOC5572942	380180400	I340V	0.05	0.42	9.44	2	AAEL010154
*CYP6AL3*		LOC5580020	60675944	V121I	0.67	0.28	7.58	1	AAEL009656
*GSTD3*		LOC5568347	301213992	A84V	0.83	0.47	7.28	1	AAEL001059
*CYP6N15*		LOC5571533	419194639	S409G	0.69	0.36	5.39	2	AAEL009122
			419194649	L412R	0.65	0.38	3.93		
*CYP6AG4*		LOC110679554	171318386	P138S	0.42	0.13	5.30	3	AAEL007010
*CYP325U1*		LOC23687635	112553718	K465R	0.63	0.90	5.28	3	AAEL017215
	*		112553724	I467T	0.61	0.89	5.23		
			112553723	T467S	0.17	0.01	3.81		
			112545541	H82Q	0.02	0.17	3.39		
			112545315	S7L	0.20	0.43	3.37		
			112545312	I6T	0.22	0.46	3.64		
aldo-keto-red		LOC5565864	337751096	M147L	0.62	0.31	5.21	2	AAEL015002
*CYP4C51*		LOC5569917	74411398	Y230C	0.82	1.00	5.18	1	AAEL008018
*CYP9M11*		LOC5571540	419111820	K340T	0.27	0.05	4.97	2	AAEL009127
*CCEunk7O*		LOC5571034	326023789	N522S	0.93	0.70	4.80	3	AAEL008757
			326024693	S260L	0.93	0.74	3.33		
*CYP9M8*		LOC5572180	357797466	L256M	0.17	0.00	4.69	2	AAEL009591
*HPX8A*		LOC5564683	235832475	L249M	0.20	0.48	4.56	1	AAEL004388
*CCEAE3O*		LOC5575613	190567112	T92A	0.99	0.80	4.50	2	AAEL011944
*GSTE1*		LOC5569844	351609764	Q84R	0.66	0.90	4.50	2	AAEL007954
*CYP9J32*	*	LOC5571141	240838981	I120V	0.62	0.33	4.43	2	AAEL008846
CYP-6A1		LOC23687976	419270865	G164E	0.00	0.15	4.32	2	AAEL017556
			419270849	N159Y	0.00	0.14	3.85		
*GPXH2*		LOC5570587	93505779	P162R	0.00	0.15	4.30	1	AAEL008397
ald-ox 6157		LOC5567568	125411491	N619S	0.21	0.02	4.27	1	AAEL006157
*CCEBE1O*		LOC5576941	42184652	G296E	0.22	0.03	4.26	3	AAEL012886
			42184685	V285A	0.25	0.06	3.69		
CYP-9f2		LOC23687786	368590144	F471L	0.62	0.87	4.23	3	AAEL017366
*CYP6N6*		LOC5571528	419242425	V425M	0.44	0.18	4.20	2	AAEL009126
*CCEjhe2O*	*	LOC5564561	388003742	M379I	1.00	0.85	4.14	2	AAEL004323
*CYP12F5*		LOC5573117	423973803	E51D	0.13	0.37	4.09	2	AAEL001960
*GSTD2*		LOC5568354	299827836	E110K	0.18	0.44	3.99	1	AAEL001078
*CYP6AK1*		LOC5565728	323189550	L491F	0.06	0.26	3.91	3	AAEL004941
ald-ox 10380		LOC5573274	182632620	I76M	0.20	0.03	3.88	2	AAEL010380
*CYP325R1*	*	LOC5567056	114802440	V4L	0.43	0.19	3.85	3	AAEL005775
*CYP6AG6*		LOC5568652	171433917	K233N	0.56	0.30	3.79	3	AAEL006992
*CYP6AG8*		LOC5579193	85685653	S499L	0.80	0.56	3.63	3	AAEL015654
*CCEglt1G*		LOC5567205	334528425	R30Q	0.72	0.47	3.47	3	AAEL000889
*GPXH3*	*	LOC5578481	160895203	C93Y	0.44	0.68	3.30	3	AAEL000495
**Recovered vs dead**					recovered	dead			
*CYP6P12v2*		LOC5576391	271406155	R247K	0.63	0.93	6.66	1	AAEL014891
			271405591	P416A	0.20	0.52	5.60		
			271406845	A17G	0.65	0.91	4.98		
*CYP6CC1*		LOC5565579	271421425	I179V	0.52	0.85	6.42	1	AAEL014890
			271421451	I170K	0.45	0.14	5.78		
			271420954	N336Y	0.67	0.92	5.12		
			271421023	A313T	0.63	0.90	5.06		
			271421665	Q99E	0.57	0.82	4.04		
*CYP6M6*		LOC5571537	419156371	E407K	0.36	0.72	6.34	2	AAEL009128
*CYP325U1*		LOC23687635	112553696	M458L	0.93	0.66	5.92	3	AAEL017215
	*		112553724	I467T	0.89	0.59	5.74		
*GPXH3*	*	LOC5578481	160895203	Y93C	0.68	0.34	5.72	3	AAEL000495
CYP-6A1		LOC23687976	419271436	M354I	0.06	0.31	4.96	2	AAEL017556
			419271427	M351I	0.05	0.28	4.67	2	
*CCEglt2G*		LOC5567206	334516359	C6F	0.89	0.62	4.77	3	AAEL000862
			334516356	A7V	0.88	0.64	4.04		
gst-1		LOC5568383	241679156	S68F	0.57	0.29	4.26	3	
			241679189	I57T	0.44	0.71	3.76		
			241679148	P71S	0.55	0.29	3.64		
*CYP325R1*	*	LOC5567056	114802440	V4L	0.19	0.45	4.24	3	AAEL005775
*CYP6AG7*		LOC5568653	171418687	F239L	0.34	0.48	4.16	3	AAEL006989
*CCEjhe2O*	*	LOC5564561	388003742	M379I	0.85	1.00	4.14	2	AAEL004323
*CYP6S3*		LOC5571531	419219864	Y347N	0.24	0.51	3.98	2	AAEL009120
			419220227	A226S	0.24	0.05	3.92	2	
*CYP9J15*		LOC5579926	368428360	T4I	0.22	0.04	3.96	3	AAEL006795
*CYP302A1*		LOC5574850	37194734	L162F	0.06	0.26	3.96	2	AAEL015655
aldo-keto-red		LOC5571546	418787288	L87V	0.25	0.06	3.85	2	
*CYP9J32*	*	LOC5571141	240838981	I120V	0.33	0.60	3.84	2	AAEL008846
*CYP12F6*		LOC5573113	423984371	S128N	0.18	0.02	3.82	2	AAEL002005
			423984353	E122G	0.18	0.02	3.81		
*CYP4D24*		LOC5569665	152855310	N296D	0.76	0.51	3.82	3	AAEL007815
ald-ox 10382		LOC5573295	182981518	A353S	0.66	0.88	3.81	2	AAEL010382
*CYP9J20v2*		LOC5564757	368529951	S43G	0.51	0.76	3.76	3	
*CYP9J19*		LOC5579933	368517592	A56V	0.22	0.04	3.50	3	AAEL006810
*CYP325M3*		LOC5576781	111696906	E292D	0.13	0.00	3.30	3	AAEL012765
**kdr vs dead**					kdr	dead			
*CCEunk7O*		LOC5571034	326025231	A81T	0.44	0.09	7.99	3	AAEL008757
*CYP6CC1*		LOC5565579	271421665	Q99E	0.45	0.82	7.20	1	AAEL014890
			271421425	I179V	0.50	0.85	7.19		
			271421536	A142T	0.58	0.21	6.98		
			271421451	I170K	0.46	0.14	6.22		
*HPX8C*		LOC5564684	235812974	I87V	0.65	0.27	7.02	1	AAEL004386
*CCEglt4H*		LOC5567220	332923424	N505S	0.92	0.61	6.55	3	AAEL000898
			332923434	S502A	0.92	0.65	5.55		
*GSTD3*		LOC5568347	301213992	A84V	0.83	0.49	6.52	1	AAEL001059
*CYP6N6*		LOC5571528	419242714	E347D	0.40	0.10	6.19	2	AAEL009126
			419242211	R496Q	0.59	0.29	4.88		
*CYP6BB2*		LOC5565578	271330273	S15T	0.44	0.78	5.91	1	AAEL014893
*CYP6P12v2*		LOC5576391	271406845	A17G	0.62	0.91	5.79	1	AAEL014891
CYP-9f2		LOC23687786	368589360	R210Q	1.00	0.80	5.73	3	AAEL017366
			368589389	E220K	0.98	0.77	5.32		
			368589361	H210Q	1.00	0.82	5.10		
			368589364	D211E	1.00	0.83	4.77		
			368589380	N217H	0.97	0.76	4.71		
			368589376	I215M	0.97	0.77	4.64		
			368589382	Q217H	0.98	0.80	4.39		
			368589377	H216Y	0.97	0.78	4.32		
			368589375	K215M	0.97	0.78	4.22		
			368589352	L207F	1.00	0.85	4.18		
			368589374	L215M	0.96	0.78	4.15		
			368589351	S207F	1.00	0.86	4.02		
			368589378	C216Y	0.97	0.80	3.83		
			368589348	S206C	1.00	0.87	3.81		
			368589381	R217H	0.97	0.80	3.72		
*CYP4J13*		LOC5565336	57986853	A269V	1.00	1.00	5.26	2	AAEL013555
aldo-keto red		LOC5564116	401732340	Q200K	0.91	0.65	5.19	3	AAEL004095
*CYP9J20v2*		LOC5564757	368529951	S43G	0.45	0.76	5.16	3	
*CYP6N13*		LOC5571524	419218265	G280D	0.28	0.05	5.15	2	AAEL009137
			419217654	T466S	0.54	0.25	4.51		
			419217648	S468T	0.25	0.54	4.46		
*HPX8B*		LOC5564679	235801599	R334H	0.61	0.29	5.15	1	AAEL004390
*CYP6N15*		LOC5571533	419193410	I17V	0.09	0.34	4.84	2	AAEL009122
aldo-keto red		LOC5564116	401732334	L202M	0.09	0.33	4.64	3	AAEL004095
			401732336	L201H	0.09	0.33	4.62		
*HPX6*		LOC5575607	271581636	I103M	0.60	0.88	4.55	1	AAEL011941
*CYP4D24*		LOC5569665	152861353	I13V	0.22	0.03	4.52	3	AAEL007815
*CYP9M10*		LOC5571539	419123623	V43A	0.30	0.59	4.41	2	AAEL009125
*CCEglt2G*		LOC5567206	334515295	L341*	0.50	0.22	4.36	3	AAEL000862
*CYP325S3*		LOC5575186	112638606	A10V	0.20	0.02	4.35	3	AAEL000357
*CYP9J15*		LOC5579926	368428360	T4I	0.23	0.04	4.29	3	AAEL006795
*CYP305A6*		LOC5573423	119675669	Y331H	0.48	0.75	4.13	2	AAEL002071
*CYP12F5*		LOC5573117	423973803	E51D	0.13	0.37	4.04	2	AAEL001960
*HPX8B*		LOC5564679	235800898	H118Q	0.13	0.36	4.02	1	AAEL004390
			235800944	Y134H	0.55	0.29	3.73		
*CYP6N17*		LOC5572939	380786471	V14L	0.12	0.38	3.94	2	AAEL010158
*HPX7*		LOC5564694	235765472	V555I	0.10	0.31	3.88	1	AAEL004401
*CYP325G2*		LOC5576783	111737982	Y434F	0.32	0.10	3.87	3	AAEL012766
*CYP6N12*		LOC5571529	419234743	K59R	0.48	0.22	3.87	2	AAEL009124
*CYP9J10*		LOC5564750	368504032	V508L	0.16	0.42	3.79	3	AAEL006798
aldo-keto red		LOC5565864	337751096	M147L	0.62	0.36	3.74	2	AAEL015002
			337751096	M147L	0.62	0.36	3.74		
*CYP325V1*		LOC23687556	112534260	V495A	0.71	0.92	3.71	3	AAEL017136

**Fig 4:**
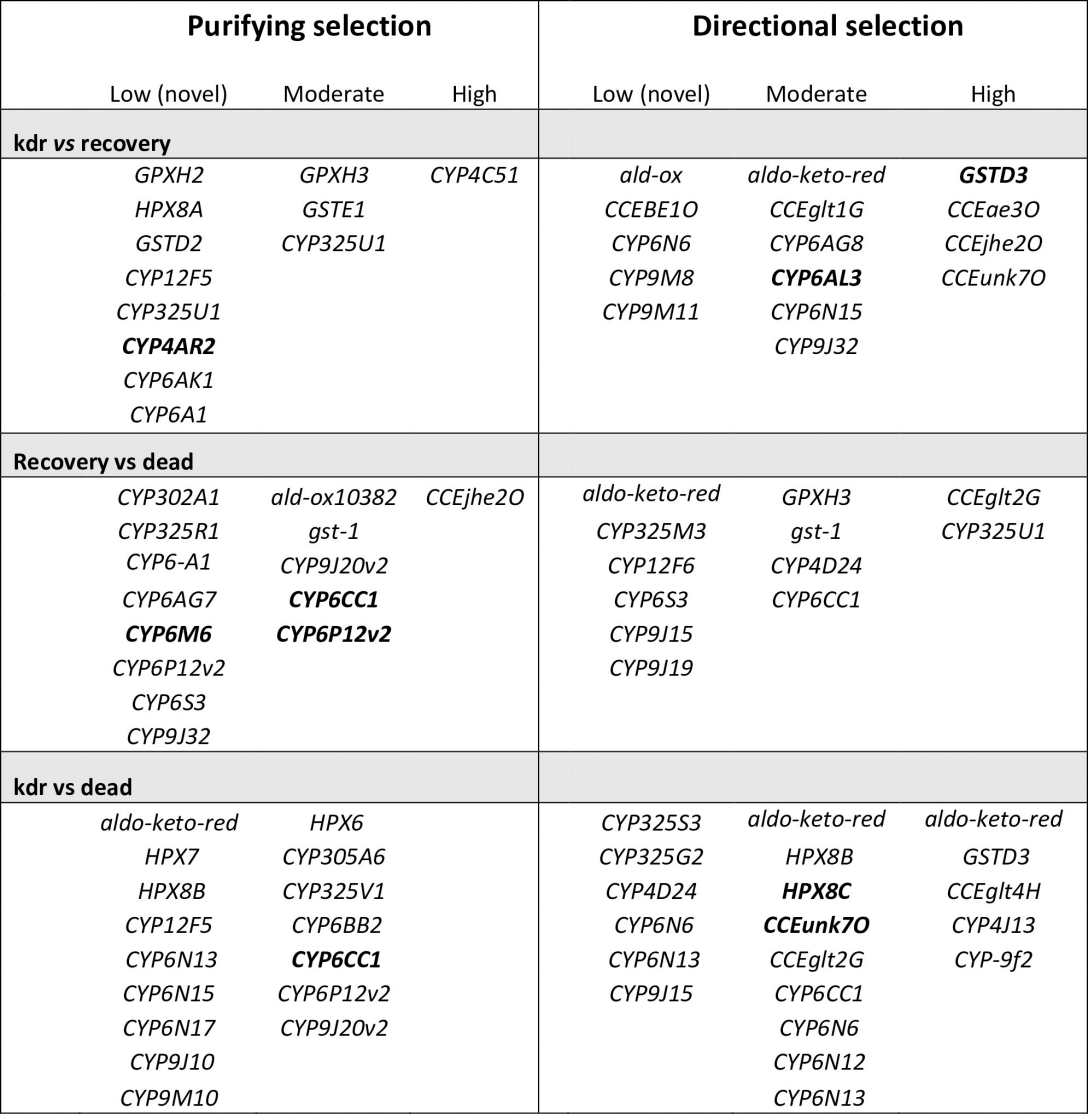
Detoxification genes under purifying or directional selection in kdr, recovered, and dead *Aedes aegypti* exposed to permethrin. We conducted three pairwise comparisons between phenotypes. The kdr vs recovery comparison involved two different resistant phenotypes. The recovery vs dead and, kdr vs dead comparisons involve one resistant and the susceptible phenotype (dead). If the frequency of the alternant SNP was higher in the resistant phenotype, we labeled the gene as directional selection. If the frequency of the alternant SNP was higher in the susceptible group, we labeled the SNP as purifying. The frequency was categorized as low (probably novel mutations) when the frequency ranged between 0 to 0.4, moderate from 0.4–0.8 and high 0.8 to 1.0. The detoxification genes with the highest LOD values for each comparison are highlighted in bold.

In the kdr vs recovered pairwise comparison, SNPs in which the alternate nucleotide frequency was higher in the kdr group were assumed to be potentially beneficial in the presence of insecticides and therefore under directional selection. In contrast, those SNPs where the alternate nucleotide frequency was higher in the recovered group were suspected to be nonbeneficial for the kdr phenotype and under neutral or purifying selection. Fig 4 shows the detoxification genes under directional and purifying selection. The frequency of the alternate allele was categorized as low (probably novel mutations) when the frequency ranged from 0 to 0.4, moderate from 0.4 to 0.8, and high from 0.8 to 1.0. Example of genes under directional selection for kdr include *GSTD3*, *CCEae3O*, *CCEunk7O*, and *CCEjhe2O*, whereas *CYP4C51* had high frequency in the recovered group, suggesting that this SNP is under purifying selection. In the recovered vs dead group, SNPs in which the alternate nucleotide frequency was higher in the recovered group were assumed to provide a beneficial protection when no *kdr*-mutations are present and therefore under directional selection. Sites where the alternant SNP was higher in the dead group were assumed to be under purifying selection.

### SNPs that differ between recovered and dead

During exposure to permethrin, 86% (343 out 401) of the mosquitoes experienced knockdown. In this knockdown group, 37% recovered (130 out of 343), whereas 62% died (213 out of 343). When comparing the genomes of the recovery and dead phenotypes, we identified 11,472 SNPs that differed significantly (2,713, 4,293 and 4,466 on chromosomes 1, 2, and 3, respectively).

### Top nonsynonymous SNPs associated with recovery

The nonsynonymous SNPs associated with recovery are shown in S2 Table. In chromosome 1, we identified 217 nonsynonymous located in 161 genes. The SNP with the highest LOD was a Y309C in putative inositol mono-phosphatase 3 (LOC110677190, LOD = 12.4), followed by a R435Q in the uncharacterized LOC5564494 (LOD = 11.6), and a L505V in uncharacterized protein F54F2.9 (LOC5579009, LOD = 11.34). In chromosome 2, 388 nonsynonymous mutations in 279 genes were identified (S2 Table). The highest LOD was a N349D in the zinc finger protein 583-like gene (LOC110677088, LOD = 12.47). The following highest SNPs consisted of L44F in transmembrane protein 231 (LOC5574513, LOD = 12.45) and V5M in uncharacterized LOC23687531 (LOD = 12.06). In chromosome 3, 403 replacements in 279 genes were associated with recovery. The SNP with the highest association was a I145T in a zinc transporter ZIP1 (LOC5578569, LOD = 13.82), followed by a P4L in general transcription factor IIF subunit 1 (LOC5563781, LOD = 12.73) and S45P in a general odorant-binding protein 45-like (LOC5566895, LOD = 11.2).

### Insecticide-target site SNPs associated with recovery

We identified 88 SNPs in 22 genes in this category (Table 2). Six nonsynonymous SNPs were found in four genes, including the acetylcholine receptor subunit alpha-like (LOC5575838, LOD = 6.7), *GPRGBB3* (LOD = 7.6 and 7.1) and the voltage-dependent calcium channel type A subunit alpha-1 (*VGCC*, LOC5564339) with an LOD of 10.9. The S679T and V410L at *VGSC* had LODs of 3.8 and 4.6, respectively.

### Cuticle SNPs associated with recovery

Approximately 112 SNPs located across 50 genes were identified in this category. The 12 nonsynonymous mutations were located in seven genes (Table 3). The highest LOD occurred in the larval cuticle protein A2B (LOC5577372, LOD = 8.8 and 6.2) and endocuticle structural protein SgAbd-6 (LOC5570308, LOD = 5.8) in chromosome 3.

### Detoxification SNPs associated with recovery

A total of 280 SNPs categorized in 103 detoxification-associated genes were associated with recovery. The 36 nonsynonymous SNPs were located in 22 genes, with LODs ranging from 3.3 to 6.6 (Table 4). In chromosome 1, the highest association occurred in *CYP6P12v2* (LOC5576391, LOD = 6.6) and *CYP6CC1* (LOC5565579, LOD = 6.4). In chromosome 2, the highest association with recovery occurred in *CYP6M6* (LOC5571537, LOD = 6.34) and CYP6-A1 (LOC23687976, LOD = 4.9 and 4.6). In chromosome 3, the highest association occurred in *CYP325U1* (LOC23687635, LOD = 5.9 and 5.74) and *GPXH3* (LOC5578481, LOD = 5.72). Fig 4 shows that *CYP6CC1*, *CYP6P12v2*, *CYP9J32*, and *CYP6M6* had SNPs under purifying selection, whereas *CYP325U1*, *CCEglt2G*, *CYP4D24*, *GSTS-1*, and *GPXH3* were under moderate to high directional selection. Novel SNPs under directional selection occurred in *CYP9J15* and *CYP9J19*.

### SNPs that differ between kdr and dead

This comparison identified SNPs that would be hypothetically selected if kdr were the only mechanism under selection, ignoring recovery as a mechanism of survival. A total of 12,381 SNPs were associated with kdr. In chromosome 1, the nonsynonymous mutations with the highest LOD values were uncharacterized LOC5576488, nose-resistant to fluoxetine protein 6 (LOC5576756), and protein msta (LOC5579017) with LOD values of 15.1, 13.1, and 11.1, respectively (S3 Table). In chromosome 2, vitamin K-dependent gamma-carboxylase (LOC110675715, LOD = 14.1) and a fatty acid synthase (LOC5573930, LOD = 13.3) had the highest association with kdr. In chromosome 3, the highest LODs occurred in the two nonsynonymous mutations on *VGSC* (S679T and V410L), with LODs of 26.1 and 25.7, respectively. These mutations were followed by two nonsynonymous mutations in a membrane-bound transcription factor site-2 protease (LOC5580236), with LODs of 24.02 and 17.5, respectively. Additional target site associations occurring in this comparison were located in *GPRGBB3* (LOC5569525, LODs = 7.13 and 7.62) and *ACE* (LOC5570776, LOD = 5.3). Table 2 shows the seven genes with nonsynonymous mutations, with LODs ranging from 3.97 to 7.93.

In the cuticle category, approximately 138 SNPs located across 49 genes were identified. The 13 nonsynonymous mutations were located in twelve genes (Table 3). The highest LOD occurred in the adult cuticle protein 1 (LOC5573913, LOD = 6.58), endocuticle SgAbd-6 (LOC5570308, LOD = 5.8) and cuticle protein CP14.6 (LOC5575766, LOD = 5.23).

In the detoxification category, 55 SNPs in 29 genes had nonsynonymous mutations with LODs ranging from 3.7 to 7.9. In chromosome 1, the highest association was *CYP6CC1* (LOC5565579, LOD = 7.2, 7.1, and 6.9), *HPX8C* (LOC5564684, LOD = 7.0), and *GSTD3* (LOC5568347, LOD = 6.5). In chromosome 2, the highest association was *CYP6N6* (LOC5571528, LOD = 6.1), *CYP4J13* (LOC5565336, LOD = 5.2), and *CYP6N13* (LOC5571524, LOD = 5.1). In chromosome 3, the highest association was *CCEunk7O* (LOC5571034, LOD = 7.9), *CCEglt4h* (LOC5567220, LOD = 6.5) and a probable cytochrome LOC23687786 (LOD = 5.7). SNPs under high directional selection occurred in *GSTD3*, *CYP4J13*, CYP9-f2, *CCEglt4H*, and aldo-keto reductase. SNPs under purifying selection included *CYP6CC1*, *CYP6BB2*, *CYP9J20*, *CYP6P12*, and CYP325V1 (Fig 4).

## Discussion

We investigated the genomic differences between the three phenotypes discriminated after exposure to permethrin, including kdr (14.5% of total), recovery (32.4% of total), and dead (53.1% of total). We used a permethrin concentration recommended by the CDC bottle bioassay, in which knocked-down mosquitoes are recorded after 30 to 60 min from the exposure, and a mortality rate is calculated. Although, the methodology does not recommend further observations, recovery rates of 20–60% of the original knocked-down mosquitoes are commonly observed within 4 h of exposure [15, 19], suggesting that recovery might be an important phenomenon in overall survival. The biological significance of recovery in the field is not well understood. A common assumption is that knocked-down mosquitoes are likely to die due to desiccation and predation. However, under ideal environmental conditions, individuals with the recovery mechanism could be favored by sublethal exposure to insecticides provided by the poor penetration of space sprays into sheltered indoor microhabitats where the mosquito rests [20]. In this study, we assume that recovery likely contributes to overall insecticide resistance in the field. Understanding the genomic differences between mosquitoes that exhibit kdr from those that recover or die after knockdown will pinpoint possible mechanisms regulating these phenotypes.

Our genomic analysis resulted in the identification of thousands of significant SNPs associated with the phenotypes. We focused on nonsynonymous mutations under the assumption that these can confer protein changes that affect the phenotype. Our results showed that SNPs at *VGSC* had the highest association with kdr but not with recovery. Significant but moderate association occurred between recovery and SNPs in additional insecticide target site, detoxification, or cuticle genes.

The role of *VGSC* as the primary site of action for the neurotoxic effects of pyrethroids in mammals and insects has been demonstrated. However, the actions of pyrethroids on secondary targets also have been associated with toxicity, mostly with the choreoathetosis and salivation (CS) intoxication syndrome by pyrethroids type 2 [21, 22]. In our study, three nonsynonymous mutations at *VGSC* (V410L and S679T) were highly associated with the kdr phenotype but not with recovery. Additionally, nonsynonymous SNPs uniquely associated with kdr were located at the *ACE*/hydrolase (target site of organophosphate and carbamates), the G protein-activated inward rectifier potassium channel 3 (target site of novel insecticides), and the gamma-aminobutyric acid type B receptor subunit 2 (possible target site of cyclodienes). The level of association of these SNPs with the phenotype was much lower than *VGSC* but still significant.

The association between nonsynonymous mutations at *VGSC* and different levels of pyrethroid resistance has been confirmed in *Ae*. *aegypti* [8, 9]. Moreover, some mutations interact and are restricted to geographical distributions [4]. In this study, two nonsynonymous mutations were identified in the *VGSC*, including V410L and S679T (S723T following *M*. *domestica*). V410L has been associated with kdr in *Ae*. *aegypti* from Mexico and has evolved in close linkage disequilibrium with V1016I [7]. Interestingly, V410L reduces the binding of pyrethroids to VGSC in mosquitoes by itself [8], whereas V1016I does not [9, 10]. Individual genotyping at V410L in the mosquitoes used for our libraries showed strong positive association between kdr with resistant homozygous genotypes, dead with wild-type homozygous genotypes, and recovered with heterozygous genotypes. Moreover, the resistance allele frequencies (q) calculated from individual genotyping for kdr (q = 0.95), recovered (q = 0.48), and dead (q = 0.18) did not differ significantly from the genome sequencing frequencies obtained by the pooled libraries for kdr (q = 0.93), recovered (q = 0.43), and dead (q = 0.15). These results and previous observations on *kdr-*mutations in *Ae*. *aegypti* from Mexico lead us to infer that V410L segregates as a recessive allele. For example, a QTL mapping confirmed the recessive nature of the V1016I to confer permethrin resistance in *Ae*. *aegypti* from Mexico [5]. In addition, the strong linkage disequilibrium between V1016I and V410L, in which 95% of the individuals collected in 2014–2016 had resistant genotypes at both loci [7] suggests that both V1016I and V410L segregate as recessive alleles.

Additional *kdr-*associated mutations at *VGSC* include V1016I, F1534C, and S679T. The latter has low fitness and can only survive when F1534C is present [15]. Although, F1534C alone confers seven- to 14-fold resistance to pyrethroids [6], the combination of F1534C with V1016I enhanced the levels of permethrin and deltamethrin resistance in electrophysiology assays [10]. In our study, we did not identify V1016I or F1534C in our libraries for different reasons. V1016I was inconsistent between the biological replicates and therefore was eliminated in the independence test. F1534C was eliminated because C1534 is approaching fixation in Tapachula, therefore, the pipeline did not identify polymorphisms at this locus. A third nonsynonymous mutation, S679T, was strongly associated with permethrin resistance in this study. This mutation was previously identified in a deltamethrin-resistant population from Merida, Mexico. This nonsynonymous mutation corresponds to residue V723 in *M*. *domestica*, and it is probably located at the linker IS6–IIS1 in the *VGSC*. The role of S679T in pyrethroid resistance is unknown [23].

SNPs associated with recovery were in the acetylcholine receptor alpha, *GPRGBB3* and the *VGCC*. Interestingly, the P1661S mutation at *VGCC* had high association with recovery (LOD = 10.9). Although the direct binding of pyrethroids to these channels has not been demonstrated, different biochemical or electrophysiological effects in these channels indicate they might play a secondary role in the toxicity of pyrethroids [18, 22]. For example, five type 2 pyrethroids and permethrin (type 1) were potent enhancers of both calcium uptake and glutamate neurotransmitter release in rat brain synaptosomes [22, 24]. Although, biochemical and electrophysiological studies show direct effects of pyrethroids on calcium channel function *in vitro*, a causal connection between these effects and pyrethroid intoxication remains unclear [21].

GABA-receptors are a major target site of picrotoxinin, chlorinated cyclodienes, and phenylpyrazoles [21, 25]. Although some evidence exists of pyrethroid and DDT effects on insect GABA responses [26], the relative low potency and incomplete stereospecificity of pyrethroids as GABA receptor antagonists in functional assays do not support a significant role in the production of pyrethroid intoxication [21]. Whether the presence of nonsynonymous mutations in the *GPRGBB3* in permethrin-exposed survivors is a result of selection by pyrethroids or other classes of insecticides requires further research.

Metabolism of insecticides is a major mechanism of resistance in *Ae*. *aegypti*. A common model of metabolic resistance assumes that the survival of mosquitoes exposed to insecticides results from enhanced metabolism (or sequestration), preventing the insecticide from reaching its target site [27]. In addition to this barrier, survival will depend on target site mutations that prevent the insecticide from exerting its toxic effects. Following the assumptions of this model, our bioassay showed that 85% of the mosquitoes were knocked down by permethrin, suggesting that metabolism was not a major mechanism to prevent intoxication of the target site. Of the 15% resistant to knockdown (kdr), 90% were homozygous resistant for the V410L mutation. The identification of metabolic mechanisms that prevent the insecticide from reaching its target site—as the model assumes—would be found in heterozygous and wild-type homozygous individuals in the kdr group (8% of the kdr mosquitoes were heterozygotes; 0% were wild-type homozygotes). We did not use this group of mosquitoes for comparisons because small sample sizes prevented us from completing pools of 25 individuals. But future experiments that include this group could decipher this model of metabolism resistance.

By using the permethrin discriminating concentration of 15 μg, mosquito survival was mostly explained by recovery (32.4%) and then by kdr (14.5%) in the mosquito population from Tapachula. Whether these responses are dose-dependent need to be addressed in future studies. Interestingly, 80% of the recovered mosquitoes were heterozygotes for the V410L locus in *VGSC*. This result suggests that carrying a single V410L allele does not protect a mosquito against knockdown but favors recovery once the pyrethroid dissociates from the ion channel and is metabolized and excreted. Therefore, the rates of recovery would be conferred by metabolism and detoxification mechanisms occurring between 1 and 4 h after insecticide exposure. Approximately 41 nonsynonymous SNPs that differ in the kdr vs recovery comparison and 36 that differ in the recovery vs dead comparison were identified. Except for five SNPs that overlapped in both comparisons (*CYP9J32*, *CCEjhe2o*, *CYP325U1*, *GPXH3*, and *CYP325R1*), unique genes were associated with kdr and recovery, suggesting different genes might explain differences between these phenotypes. Genes associated with *kdr* included three esterases (*CCEae3O*, *CCEaeB1*, and *CCEunk7O*); seven *CYP6*; three *CYP325; CYP4J;* and four redox, two delta and one epsilon GSTs. Interestingly, *CCEunk7O* was previously associated with kdr following exposure to permethrin in a previous QTL mapping study in *Ae*. *aegypti* from Mexico [5]. Because *CCEunk7O* is close to *VGSC* in the AaegL5 genome assembly, this association may be the result of a genetic sweep, as was observed recently in a study where several genes close to *VGSC* were highly associated with deltamethrin resistance [23]. The same study also identified SNPs at *CCEae30*, *CCEaeB1*, *CYP325*, and *CYP4J* in association with deltamethrin resistance in Merida, Mexico [19]. Additionally, *CYP4J* and *CYP325* genes have been upregulated in pyrethroid resistant *Ae*. *aegypti* from the United States, Mexico, Vietnam, and Thailand [11, 28–30]. Specifically, in *Ae*. *aegypti* from Mexico, the upregulation of *CYP325G3*, *CYP4J13*, *CYP6NAE1*, *GSTE2*, and other genes was selected for after one generation of artificial selection with permethrin [29].

Specific genes associated with recovery included five *CYP9J (-2*, *15*, *20*, *26*, *and 29*), seven *CYP6* (including *CYP6P12*, *CYP6BB2*, *CYP6Z9*, and *CYP6M6*), and four delta and two theta GSTs. Similarly, the overexpression of several of these genes has been reported in insecticide resistant *Ae*. *aegypti* from Malaysia [31], Laos [32], Mexico [33] and Thailand [11, 29]. Moreover, the functional metabolism of pyrethroids has been demonstrated for *CYP9J26*, *CYP9J28*, *CYP9J32*, and *CYP6BB2* in *Ae*. *aegypti* [34, 35] and *CYP6P12* in *Ae*. *albopictus* [36].

Enhanced metabolism can result from two different mechanisms: gene overexpression or allele variants conferring higher catalytic properties to the enzymes coded by these genes. To date, most studies have focused on expression analysis between resistant and laboratory susceptible strains, but a few studies have identified allele variants associated with resistance. For example, in *CYP6P9A* and *CYP6P9B* from *An*. *funestus*, a single allele was associated with pyrethroid resistance [37]. In addition, allele variants have been associated with temephos resistance in *Ae*. *aegypti* from Brazil or Thailand [38]. Interestingly, one study compared the transcription and copy number in pyrethroid-resistant strains from different continents [39]. The study showed that detoxification genes differentially expressed in resistant populations were contained in genomic clusters affected by copy number variations associated with resistance. Positive correlation occurred in three CCEs (*CCEae3A*, *CCEae4A*, *and CCEae6A*), *CYP9J21*, *CYP9J22*, *CYP6BB2*, and *CYP6P12* [39]. In this study, we did not test for expression analysis. However, previous studies evaluated the expression profiles in Mexican *Ae*. *aegypti* colonies artificially selected for permethrin resistance [29]. The study showed that resistance ratios increased between 60- and 165-fold among strains, but this increase in resistance was associated with only slight changes in CYP expression profiles (less than three-fold). Mostly, the highest differences seemed to be constitutive [29]. Whether SNPs found in specific comparisons are directly associated with more efficient metabolism of permethrin during the first hour (kdr) or following recovery after 4 h, requires further research.

Identifying the specific genes associated with resistance phenotypes is the first step in the development of genetic SNP markers of resistance. Our results show that several detoxification genes are associated with kdr and recovery. Some genetic markers might only be artifacts of genetic sweeps or might follow different evolutionary mechanisms of selection. The applicability of this study beyond southeastern Mexican populations requires further investigation. Two studies have used exome-wide sequencing to identify SNPs associated with pyrethroid resistance in Mexico. These included two sites located less than 10 km apart in the Yucatan Peninsula (Vergel and Viva Caucel) [19, 33] and Tapachula (this study), which is ~1000 km from Yucatan. Both studies have shown a strong association between kdr and mutations at VGSC. Selection of *kdr*-mutations across geographical locations can be explained by the uniform practices of insecticide application by the Mexican vector control programs. Also, by the high gene flow in southeastern *Ae*. *aegypti* populations recorded in a large-scale mitochondrial population genetics study [40] and a small-scale SNP study in the Yucatan Peninsula [41]. Currently, *kdr*-mutations are widespread in *Ae*. *aegypti* from Mexico and the United States. Whether the same SNPs at compensatory or detoxification genes associated with insecticide resistance become selected across *Ae*. *aegypti* populations in North America remains unknown. Recent genome-wide comparisons in *Ae*. *aegypti* populations from California show large differentiation between genetic clusters due to recent introductions and from multiple genetically diverged populations [42, 43]. For example, Southern California were less genetically diverse that Northern California populations. This low diversity was likely a signature of bottlenecks caused by recent founder effects and/or vector control measures [43].

Recent studies have shown the importance of reduced insecticide penetration of the cuticle as a mechanism of resistance in mosquito vectors. In *Anopheles gambiae*, thickening of the cuticle was associated with reduced permeability to pyrethroids in resistant mosquitoes [44, 45]. These studies have revealed that the basis of cuticular thickening is quantitative changes in the composition of the cuticle. Furthermore, the role of certain enzymes (*CYP4G16*, *CYP4G17*) in enhancing the biosynthesis of epicuticular hydrocarbons and the upregulation of cuticular genes in resistant strains has been demonstrated. So far, 293 cuticle proteins (CPR) have been characterized in *Anopheles gambiae* [46], and at least 300 are present in the recently annotated *Ae*. *aegypti* genome assembly. Our study identified nonsynonymous mutations in several CPR genes; however, three genes had high LODs associated with kdr (LOC5571160 and LOC5571167) and recovery (LOC5577598). Further studies are required to test how these qualitative changes in cuticle proteins are associated with pyrethroid resistance in *Ae*. *aegypti* field populations.

## Conclusion

Pyrethroid resistance in *Ae*. *aegypti* threatens our ability to control arboviral diseases. Understanding the mechanisms of pyrethroid resistance and the interactions and evolution of that resistance will be necessary to develop diagnostic tools to support insecticide management strategies. Two phenotypes—kdr and recovery—are involved in pyrethroid survival. This study identified mutations at *VGSC* controlling kdr but not recovery. Additional target site SNPs in the *VGCC* and GABA receptor genes were associated with recovery. Additionally, some specific detoxification genes were uniquely associated with kdr or with recovery. Understanding the role of these genes in the metabolism of pyrethroid will increase our knowledge of the evolution of resistance mechanisms in the field.

## Methods

### Mosquito colony and bioassays

We used a field mosquito colony named Colinas, which was collected in 2017 from Tapachula, Chiapas, Mexico (N 14’ 55” 43.6, W 92’ 14” 58.8). Briefly, we collected larvae from patios of approximately 25 houses in this neighborhood. Approximately 1,000 larvae were transferred to the Insectary at Centro Regional de Investigaciones en Salud Publica. Emerged mosquitoes were identified as *Ae*. *aegypti* and bloodfed to produce the F_1_ offspring. Mosquito F1-egg papers were shipped to Colorado State University, where we performed the bottle bioassay on emerged adult mosquitoes to establish the levels of permethrin resistance relative to the New Orleans susceptible reference strain. We exposed approximately 75 mosquitoes to five different concentrations of permethrin for 1 h. Then, mosquitoes were removed from the treated bottle and were kept on a holding cup to score the mortality at 24 hours. Permethrin concentrations ranged from 0.1–1.5 μg/bottle for New Orleans and 7–50 μg/bottle for Colinas. Knockdown and mortality was scored at 1 or 24 h of observation, respectively. Following a binomial regression model and using the IRMA quick calculator [47], we calculated the lethal concentration that kills 50% of the mosquitoes. The permethrin LC_50_ for Colinas was 37.3-fold higher than that of the New Orleans susceptible strain. The LC_50_ values were 21.25 μg/bottle (95% CI = 18.5–24.4 μg) for Colinas and 0.56 μg/bottle (95% CI = 0.51–0.56 μg) for New Orleans.

For the genome-wide association study, we used a permethrin-discriminating concentration (LC50 = 15 μg) that allowed us to discriminate three different phenotypes. The interior walls of 10 Wheaton bottles (250 ml) were coated with 15 μg of permethrin (Sigma-Aldrich, St. Louis, Missouri) diluted in 1 ml acetone. Following the evaporation of acetone overnight, we performed the bioassay. Approximately 40 non-bloodfed female mosquitoes (3–4 days old) were aspirated into each permethrin-coated bottle. After the mosquitoes had been exposed for 1 h, we transferred active mosquitoes to a clean 1-quart cup for observation. The remaining knocked-down mosquitoes in the bottle were transferred to a second cup for observation. These cups were placed in an incubator for 4 h. At this time, we recorded our three phenotypes: 1) “knockdown-resistant” (kdr) refers to those mosquitoes still active after 1 h of insecticide exposure and that maintained activity 4 h after being transferred to clean cups; 2) “recovered” refers to those mosquitoes that were knocked-down at 1 h and became active again in the 4 h after being transferred to clean cups; and 3) “dead” those mosquitoes that were knocked down at 1 h and did not become active again in the 4 h after being transferred to clean cups (Fig 1). In this study, we compared the genome of pooled mosquitoes from the three phenotypic groups: kdr vs recovered, recovered vs dead, and kdr vs dead.

### Sample pooling and library preparation

We constructed six genomic libraries (gDNA): two for the knockdown-resistant (kdr) group, two for the recovery group, and two for the dead group. Each library consisted of pools of 25 mosquitoes. Before the individual mosquitoes were pooled, gDNA from each mosquito was quantified using the Quant-IT Pico Green kit (Life Technologies, Thermo Fisher Scientific Inc.). Approximately 40 ng of each individual DNA sample was used for a final DNA pool of 1 μg. Pooled DNA was sheared and fragmented by sonication to obtain fragments between 300–500 bp (Covaris Ltd., Brighton, UK). We prepared one library for each of the six DNA pools following the Low Sample (LS) protocol from the Illumina TrueSeqDNA PCR-Free Sample preparation guide (Illumina, San Diego CA). Because 47% of the *Ae*. *aegypti* genome consists of transposable elements and other forms of repetitive DNA [48], we performed an exome-capture hybridization to enrich for coding sequences using custom SeqCap EZ Developer probes (NimbleGen, Roche). Probes covered protein coding sequences (not including UTRs) in the AaegL1.3 genebuild using the exonic coordinates specified previously [49]. In total, 26.7 Mb of the genome (2%) was targeted for enrichment. TruSeq libraries were hybridized to the probes using the xGen Lock Down recommendations (Integrated DNA Technologies). The targeted DNA was eluted and amplified (10–15 cycles). Pair-end sequencing was run in a flow cell of NovaSEQ S4 and performed by the Genomics Core University of Colorado Anschutz Medical Campus (Aurora, Colo.).

### Analysis pipeline

Our analysis compared the frequency of the alternate allele at each polymorphic site between the two different phenotypes. The alternate allele consisted of an allele not present in the reference AaegL5 genome assembly. In this study, we performed three pairwise comparisons: 1) kdr vs recovered, 2) recovered vs dead, and 3) kdr vs dead.

All sequencing data generated under this project is available at the National Center for Biotechnology Information (NCBI) Sequence Read Archive, Bioproject PRJNA731165. Colinas kdr replicates 1 and 2, are submitted as SRR14609309 and SRR14609308, respectively. Colinas recovered replicates 1 and 2, are SRR14609307 and SRR14609306, respectively. Colinas dead replicates 1 and 2, are SRR14609305 and SRR14609304.The raw sequence files (*.fastq) for each pair-ended gDNA library were aligned to a custom reference physical map generated from the assembly AaegL5 [50] using the package GSNAP (Genomic Short-read Nucleotide Alignment) [51]. We chose a minimum coverage of 25 and minimum base quality of 30.

A series of R scripts were used to split the genomic sites in the three chromosomes. A list of common sites within biological replicates and common sites between the phenotypes was then generated. The following script selected polymorphic sites (SNPs) and generated tables for allele counts in each of the four libraries (e.g., kdr1, kdr2, dead1, dead2). Allele frequencies for the alternant allele were calculated for each phenotype, and a goodness of fit test identified SNPs with consistent proportions within replicates (*p* > 0.05). Then, we built contingency tables and calculated the heterogeneity χ^2^ with n—1 degrees of freedom to compare the proportion of the alternate allele between the phenotypes (the probability derived from this analysis was -log_10_ transformed to provide a “LOD” value). Additionally, we calculated the expected heterozygosity (*H*_*exp*_*)* of each site where $Hexp=1-\sum_{i=1}^{n}{pi}^{2}$ and *n* is the number of alternate nucleotides at a site. We applied a Benjamini-Hochberg correction for false discovery rate [18] for each chromosome separately (α = 0.01). SNPs with a LOD above the cutoff were considered significant. Each significant SNP was annotated with the position (intergenic region, gene, introns, 3’-UTR, 5’-UTR, synonymous or nonsynonymous site). This information is included in S4–S6 Tables. Finally, a Fortran Program was used to identify whether the alternate SNP conferred a nonsynonymous mutation.

Individual genotyping of the V410L at VGSC was performed in the 50 individuals included in the libraries by using melting curve analysis of the allele-specific PCR methodology described in Saavedra-Rodriguez et al. 2018 [18].

## Supporting information

S1 Table List of nonsynonymous SNPs differing between kdr and recovered *Ae*. *aegypti* mosquitoes exposed to permethrin. The gene ID, site (based in AaegL5 genome assembly), alternate nucleotide frequency in kdr, alternate nucleotide frequency in recovered, LOD (–log_10_(*p* value)), heterozygosity in kdr, heterozygosity in recovered, total heterozygosity, amino acid substitution, functional category, vector base aliases and gene description.
(XLSX)

S2 Table List of nonsynonymous SNPs differing between recovered and dead *Ae*. *aegypti* mosquitoes exposed to permethrin. The gene ID, site (based in AaegL5 genome assembly), alternate nucleotide frequency in recovered, alternate nucleotide frequency in dead, LOD (–log_10_(*p* value)), heterozygosity in recovered, heterozygosity in dead, total heterozygosity, amino acid substitution, functional category, vector base aliases and gene description.
(XLSX)

S3 Table List of nonsynonymous SNPs differing between kdr and dead *Ae*. *aegypti* mosquitoes exposed to permethrin. The gene ID, site (based in AaegL5 genome assembly), alternate nucleotide frequency in kdr, alternate nucleotide frequency in dead, LOD (–log_10_(*p* value)), heterozygosity in kdr, heterozygosity in dead, total heterozygosity, amino acid substitution, functional category, vector base aliases and gene description.
(XLSX)

S4 Table List of SNPs differing between kdr and recovered *Ae*. *aegypti* mosquitoes exposed to permethrin. The gene ID, site (based in AaegL5 genome assembly), alternate nucleotide frequency in kdr, alternate nucleotide frequency in recovered, LOD (–log_10_(*p* value)), heterozygosity in kdr, heterozygosity in recovered, total heterozygosity, reference nucleotide, alternate nucleotide, gene orientation in chromosome, SNP position in gene, amino acid substitution classification (synonymous vs replacement), alternate nucleotide position in codon, amino acid residue, reference codon, reference amino acid, alternate codon, alternate aminoacid, chromosome, vector base identification, gene description and insecticide resistance category (target, cuticle, detoxification, other).
(CSV)

S5 Table List of SNPs differing between recovered and dead *Ae*. *aegypti* mosquitoes exposed to permethrin. The gene ID, site (based in AaegL5 genome assembly), alternate nucleotide frequency in recovered, alternate nucleotide frequency in dead, LOD (–log_10_(*p* value)), heterozygosity in recovered, heterozygosity in dead, total heterozygosity, reference nucleotide, alternate nucleotide, gene orientation in chromosome, SNP position in gene, amino acid substitution classification (synonymous vs replacement), alternate nucleotide position in codon, amino acid residue, reference codon, reference amino acid, alternate codon, alternate aminoacid, chromosome, vector base identification, gene description and insecticide resistance category (target, cuticle, detoxification, other).
(CSV)

S6 Table List of SNPs differing between kdr and dead *Ae*. *aegypti* mosquitoes exposed to permethrin. The gene ID, site (based in AaegL5 genome assembly), alternate nucleotide frequency in kdr, alternate nucleotide frequency in dead, LOD (–log_10_(*p* value)), heterozygosity in kdr, heterozygosity in dead, total heterozygosity, reference nucleotide, alternate nucleotide, gene orientation in chromosome, SNP position in gene, amino acid substitution classification (synonymous vs replacement), alternate nucleotide position in codon, amino acid residue, reference codon, reference amino acid, alternate codon, alternate amino acid, chromosome, vector base identification, gene description and insecticide resistance category (target, cuticle, detoxification, other).
(CSV)
